# Supplementary material for: CD8A as a Prognostic and Immunotherapy Predictive Biomarker Can Be Evaluated by MRI Radiomics Features in Bladder Cancer
Source: Cancers (Basel). 2022 Oct 5;14(19):4866. doi: 10.3390/cancers14194866 (PMC9564077; doi:10.3390/cancers14194866)
Supplement: Supplementary file 1 [file cancers-14-04866-s001.zip › cancers-1889209-supplementary.pdf]

cancers

MDPI

Supplementary Materials: CD8A as a Prognostic and Immunotherapy Predictive Biomarker Can Be Evaluated by MRI Radiomics Features in Bladder Cancer

Zongtai Zheng, Yadong Guo, Xiongsheng Huang, Ji Liu, Ruiliang Wang and Xiaofu Qiu and Shenghua Liu

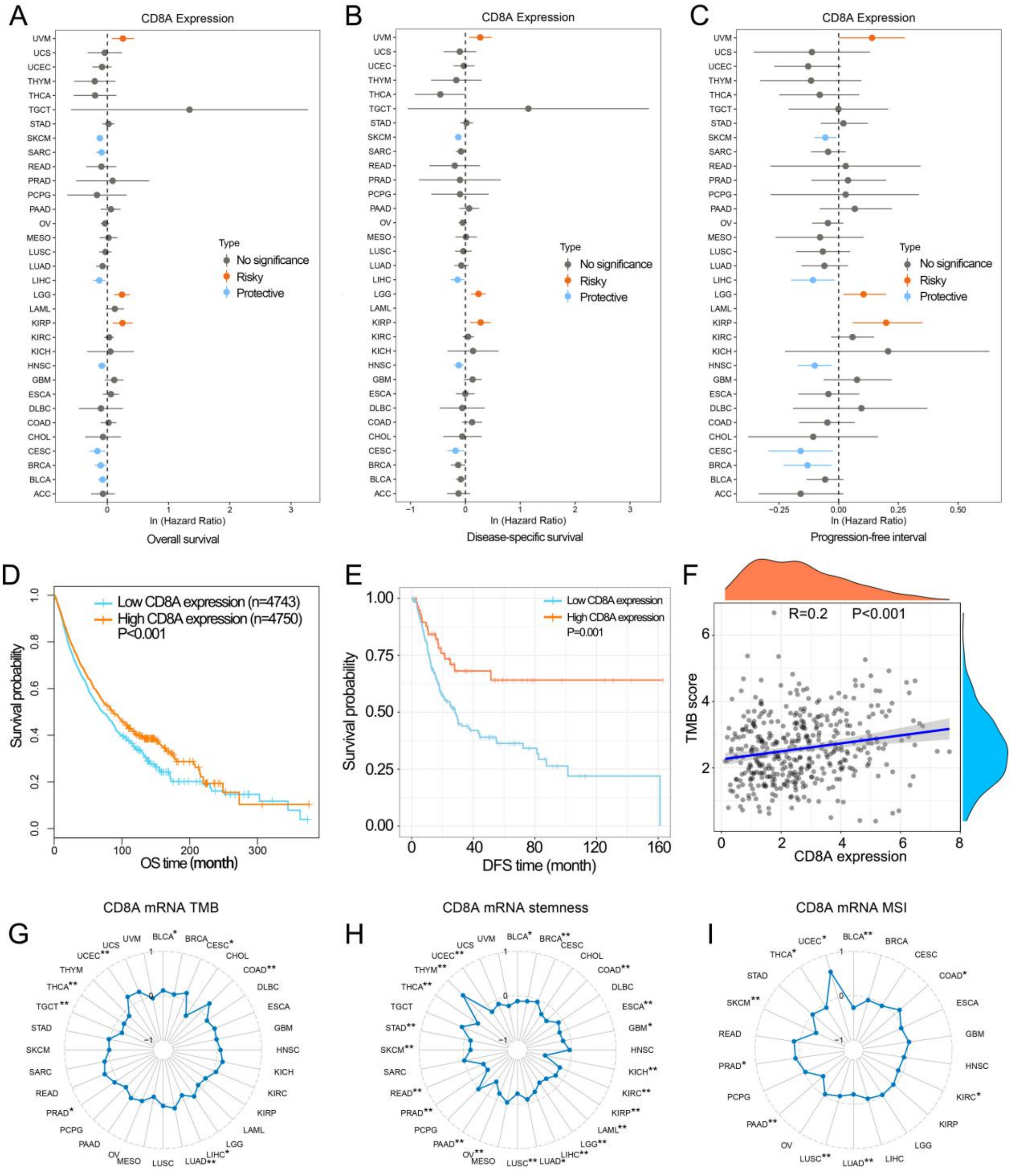

Figure S1. Pan-cancer analysis of CD8A expression. (A-C) Risk plot of correlation between CD8A with OS (A), DFS (B) and PFS (C). (D) Kaplan–Meier plots of CD8A in all tumor patients in The Cancer Genome Atlas. (E) Kaplan–Meier curves for DFS of TCGA-BCa with CD8A expression levels. (F) Correlations between CD8A expression and TMB. (G-I) Pan-cancer analysis of the association of CD8A expression with TMB, stemness and MSI. OS, overall survival; DFS: disease-specific

survival; PFS: progression-free interval; TCGA: The Cancer Genome Atlas; TMB: tumor mutation burden; MSI: Microsatellite Instability. *: P < 0.05., **: P < 0.01.

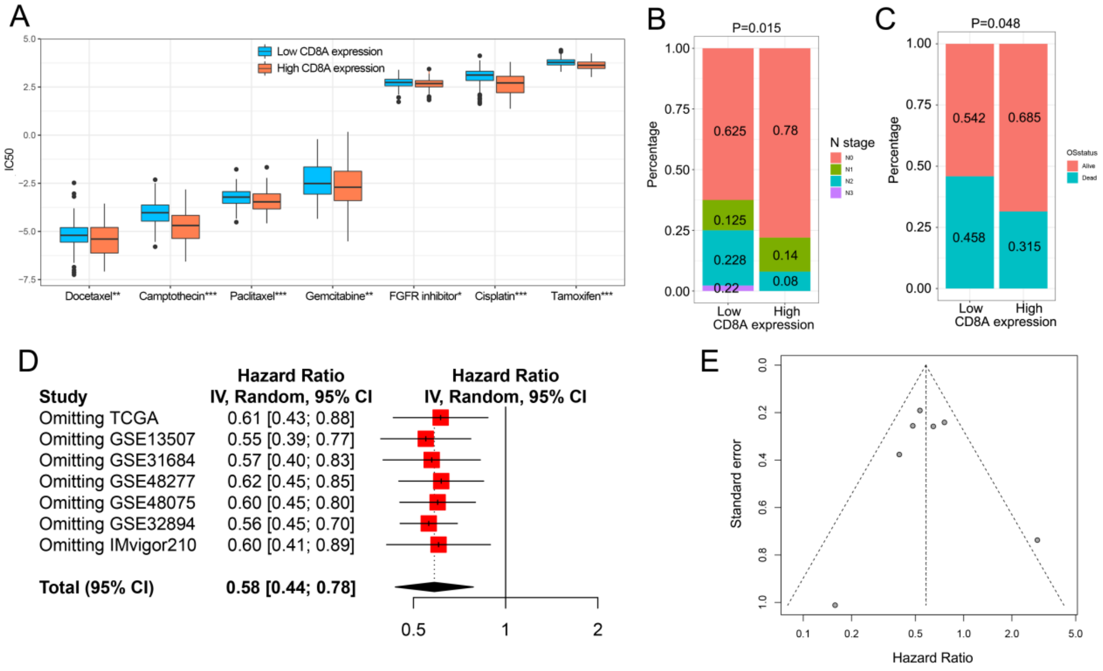

Figure S2. The association of CD8A with clinical features and drug sensitivity. (A) The IC50s values of seven drugs for anti-cancer treatment between high and low CD8A expression groups. (B) Percentages of the N stage between high or low CD8A expression groups in TCGA. (C) Percentages of the survival outcomes between high or low CD8A expression groups in TCGA. (D) Sensitivity analysis of the meta-analysis. (E) Funnel plot of the meta-analysis. CI: confidence interval; OS: overall survival. *: P < 0.05., **: P < 0.01, ***: P < 0.001.

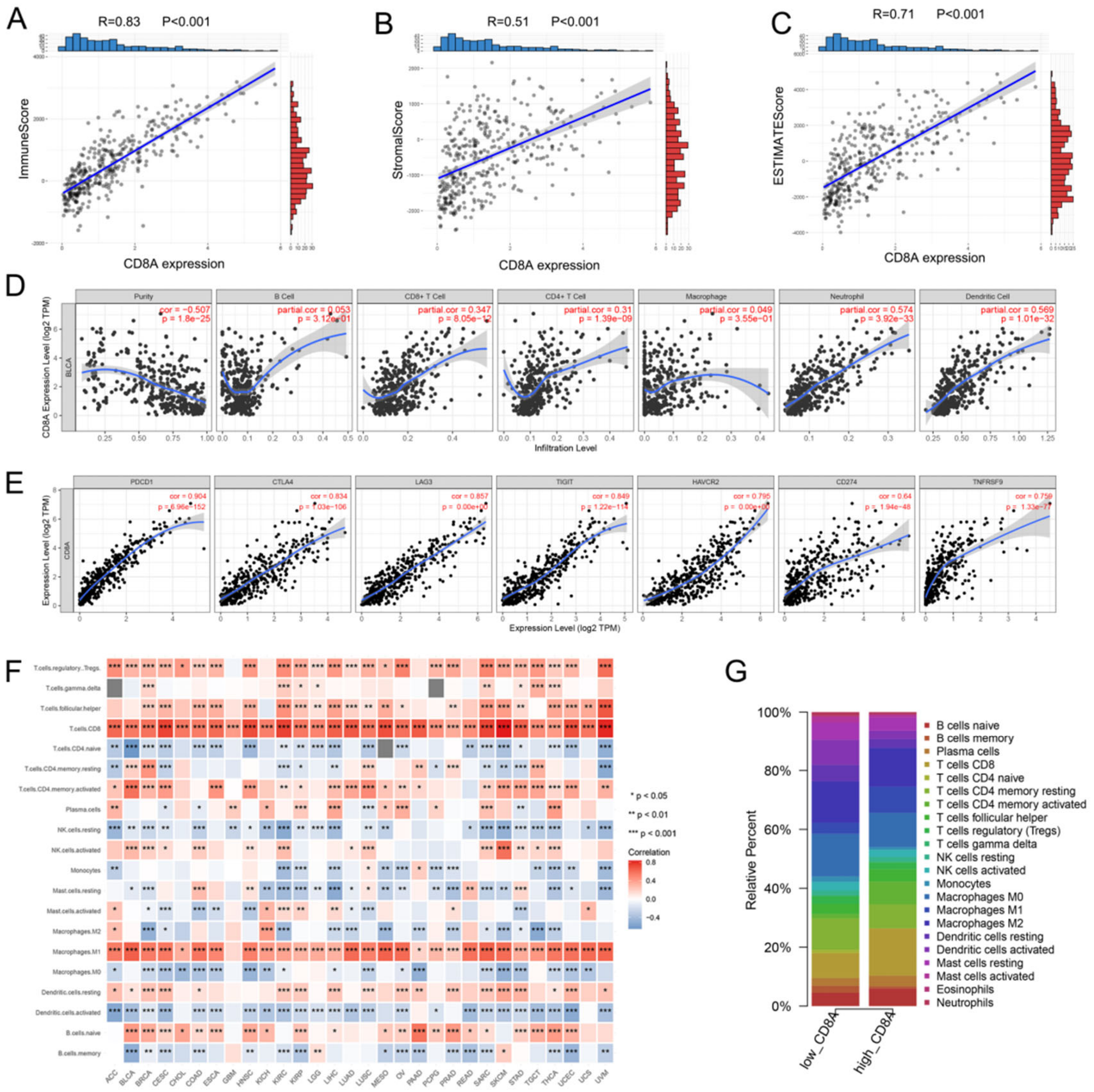

Figure S3. The association of CD8A with TME characteristics. (A-C) The association of CD8A with ImmuneScore (A), StromalScore (B) and ESTIMATEScore (C). (D) The association between CD8A and TIICs. (E) The association between CD8A and critical immune checkpoint genes. TIICs, Tumor-infiltrating immune cells. (F) Pan-cancer analysis of the association of CD8A expression with TIICs. (G) The relative infiltration levels of TIICs based on the expression of CD8A. TME: tumor microenvironment; TIICs: tumor-infiltrating immune cells. *: P < 0.05., **: P < 0.01, ***: P < 0.001.

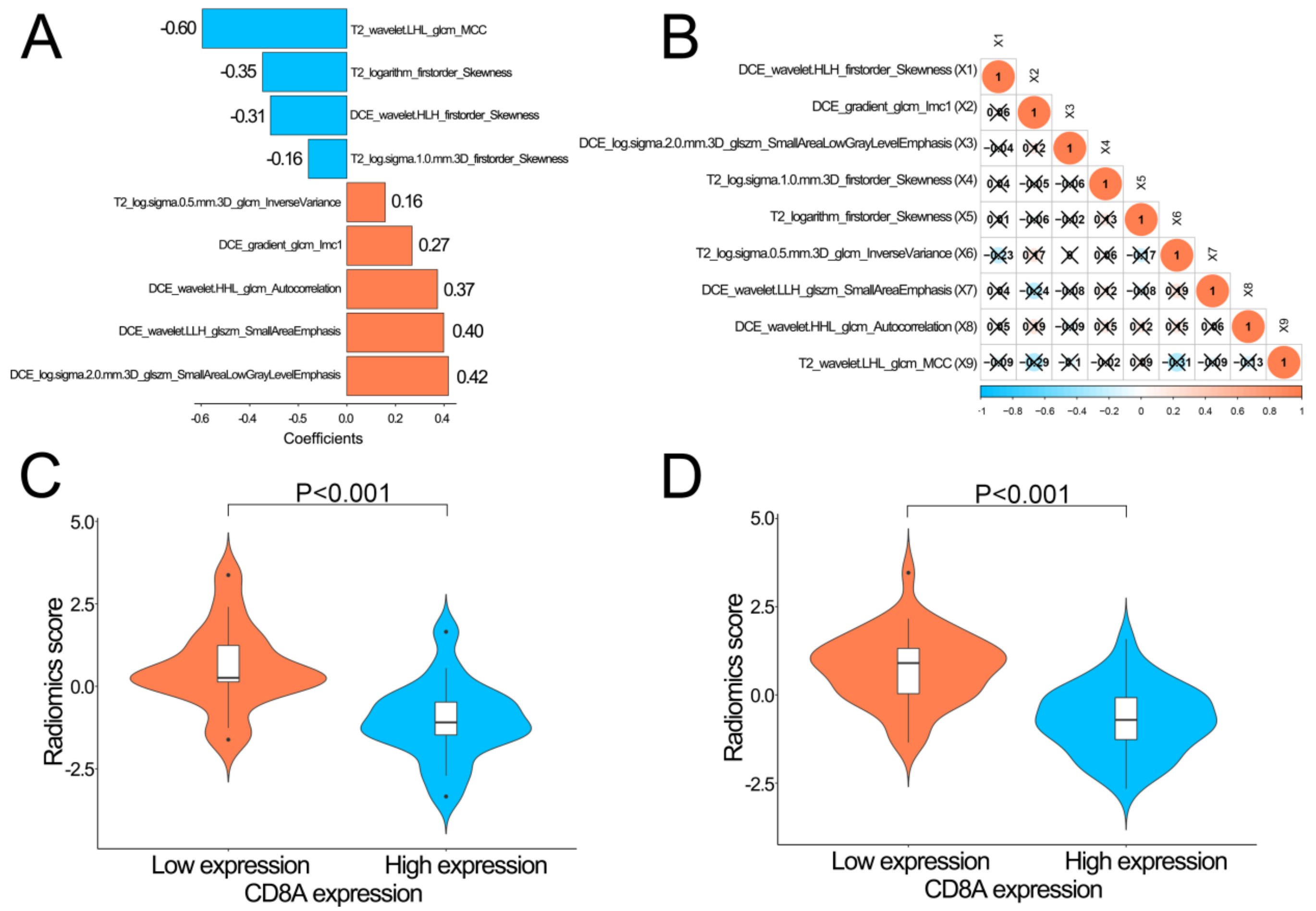

Figure S4. The Correlation matrix and coefficients of the radiomics features in LASSO model. (A) The associations between nine radiomics features. (B) The coefficients of nine radiomics features. (C-D) The violin plots of the LASSO model in the training (C) and validation sets (D). LASSO: least absolute shrinkage and selection operator. LASSO: least absolute shrinkage and selection operator.

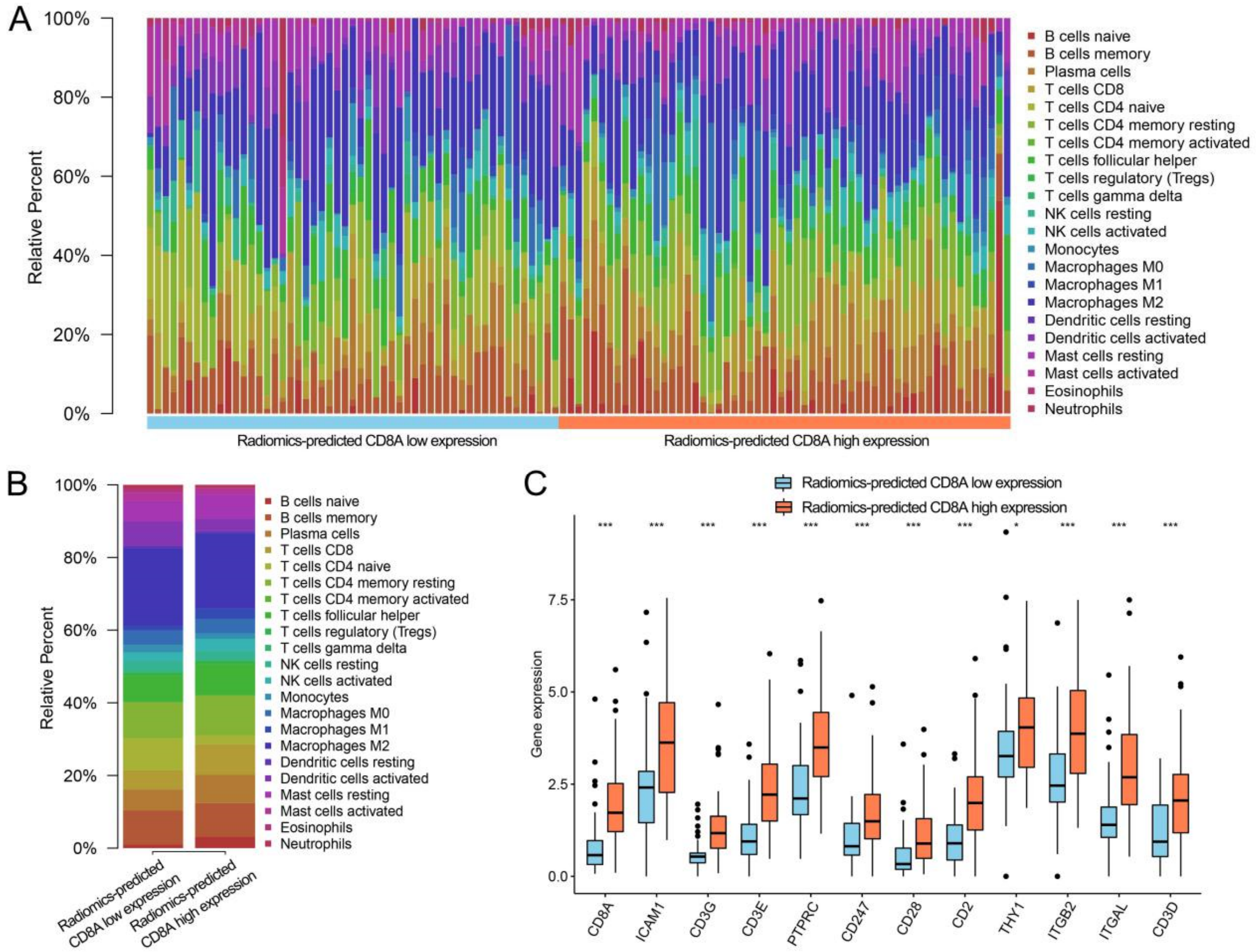

Figure S5. The association of radiomics-predicted CD8A expression with TIICs and T cytotoxic pathway-related genes in our center. (A) The proportions of 22 TIICs in each sample quantified by CIBERSORT algorithm in our center. (B) The relative infiltration levels of TIICs based on the radiomics-predicted CD8A expression. (C) The difference of the expression of T cytotoxic pathway-related genes between radiomics-predicted high and low CD8A expression. TIICs: tumor-infiltrating immune cells. *: P < 0.05., **: P < 0.01., ***: P < 0.001.

Table S1. Data set information included in this study.

Accession number	Number of patients with prognostic information	Tumor type	Platform	Method
GSE13507	165	Bladder cancer	GPL6102	Illumina human-6 v2.0 expression beadchip
GSE31684	93	Bladder cancer	GPL570	Affymetrix Human Genome U133 Plus 2.0 Array
GSE32894	308	Bladder cancer	GPL6947	Illumina HumanHT-12 V3.0 expression beadchip
GSE48075	73	Bladder cancer	GPL6947	Illumina HumanHT-12 V3.0 expression beadchip
GSE48277	73	Bladder cancer	GPL14951	Illumina HumanHT-12 WG-DASL V4.0 R2 expression beadchip
IMvigor210C	348	Metastatic urothelial cancer	-	TruSeq RNA Access technology
GSE93157	65	Non-small cell lung carcinoma, head and neck squamous cell carcinoma and melanoma	GPL19965	nCounter PanCancer Immune Profiling Panel

Table S2. The relative infiltration levels of TIICs based on the expression of CD8A.

Type of immune cell	CD8A low expression	CD8A high expression
B cells naive	0.045186251	0.059736411
B cells memory	0.024045313	0.007746776
Plasma cells	0.026539202	0.035955359
T cells CD8	0.084594035	0.16023612
T cells CD4 naive	0.010613112	0.000733953
T cells CD4 memory resting	0.106566973	0.081099648
T cells CD4 memory activated	0.014846028	0.077344485
T cells follicular helper	0.034729393	0.041834215
T cells regulatory (Tregs)	0.026034636	0.0226807
T cells gamma delta	0.004244008	0.004313924
NK cells resting	0.015730837	0.01530847
NK cells activated	0.029023116	0.023957135
Monocytes	0.018013098	0.008947608
Macrophages M0	0.145128955	0.116547658
Macrophages M1	0.037131605	0.088241944
Macrophages M2	0.141029636	0.132460834
Dendritic cells resting	0.055176501	0.029719086
Dendritic cells activated	0.085219803	0.030343905
Mast cells resting	0.060498376	0.045092784
Mast cells activated	0.019413601	0.008849456
Eosinophils	0.005379101	0.001526306
Neutrophils	0.010856422	0.007323221

Table S3. The relative infiltration levels of TIICs based on the radiomics-predicted CD8A expression status.

Type of immune cell	Radiomics predicted-CD8A low expression	Radiomics predicted-CD8A high expression
B cells naive	0.009902106	0.030880178
B cells memory	0.094028475	0.0927528
Plasma cells	0.057409397	0.078116559
T cells CD8	0.053307302	0.083843239
T cells CD4 naive	0.087377404	0.02505377
T cells CD4 memory resting	0.09903117	0.108408124
T cells CD4 memory activated	0.000411267	0.002584007
T cells follicular helper	0.072785295	0.083611706
T cells regulatory (Tregs)	0.008943013	0.011428479
T cells gamma delta	0.003130909	0.000147586
NK cells resting	0.028434929	0.026287422
NK cells activated	0.023421794	0.033034609
Monocytes	0.02138773	0.015637255
Macrophages M0	0.039248586	0.039286633
Macrophages M1	0.012700686	0.028208053
Macrophages M2	0.213240149	0.207674038
Dendritic cells resting	0.007860665	0.005551927
Dendritic cells activated	0.066583596	0.034129038
Mast cells resting	0.055669276	0.066575796
Mast cells activated	0.024125286	0.015544255
Eosinophils	0.010911254	0.003902536
Neutrophils	0.010089711	0.00734199
